# Increased expression of NF-AT3 and NF-AT4 in the atria correlates with procollagen I carboxyl terminal peptide and TGF-β1 levels in serum of patients with atrial fibrillation

**DOI:** 10.1186/1471-2261-14-167

**Published:** 2014-11-25

**Authors:** Fei Zhao, ShiJiang Zhang, YiJiang Chen, WeiDong Gu, BuQing Ni, YongFeng Shao, YanHu Wu, JianWei Qin

**Affiliations:** Cardiothoracic Surgery Department, First Affiliated Hospital of Nanjing Medical University, 300 Guangzhou Road, Jiangsu Province Nanjing, 210029 China

**Keywords:** Atrial fibrillation, Atrial fibrosis, Transcription factor, NF-AT3, NF-AT4, Carboxyl terminal peptide from pro-collagen I, N-terminal type I procollagen propeptides, N-terminal type III procollagen propeptides

## Abstract

**Background:**

Atrial fibrillation (AF) is the most common cardiac arrhythmia in clinical practice. Unfortunately, the precise mechanisms and sensitive serum biomarkers of atrial remodeling in AF remain unclear. The aim of this study was to determine whether the expression of the transcription factors NF-AT3 and NF-AT4 correlate with atrial structural remodeling of atrial fibrillation and serum markers for collagen I and III synthesis.

**Methods:**

Right and left atrial specimens were obtained from 90 patients undergoing valve replacement surgery. The patients were divided into sinus rhythm (n = 30), paroxysmal atrial fibrillation (n = 30), and persistent atrial fibrillation (n = 30) groups. NF-AT3, NF-AT4, and collagen I and III mRNA and protein expression in atria were measured. We also tested the levels of the carboxyl-terminal peptide from pro-collagen I, the N-terminal type I procollagen propeptides, the N-terminal type III procollagen propeptides, and TGF-β1 in serum using an enzyme immunosorbent assay.

**Results:**

NF-AT3 and NF-AT4 mRNA and protein expression were increased in the AF groups, especially in the left atrium. NF-AT3 and NF-AT4 expression in the right atrium was increased in the persistent atrial fibrillation group compared the sinus rhythm group with similar valvular disease. In patients with AF, the expression levels of nuclear NF-AT3 and NF-AT4 correlated with those of collagens I and III in the atria and with PICP and TGF-β1 in blood.

**Conclusions:**

These data support the hypothesis that nuclear NF-AT3 and NF-AT4 participates in atrial structural remodeling, and that PICP and TGF-β1 levels may be sensitive serum biomarkers to estimate atrial structural remodeling with atrial fibrillation.

## Background

Atrial fibrillation (AF) is the most common cardiac arrhythmia in clinical practice [1, 2]. Valvular heart disease (VHD), which comprises pathological changes in the mitral or aortic valves, causes AF. Valvular surgery is an option to prevent heart failure in serious VHD patients [3, 4]. AF is self-perpetuating because tachyarrhythmia causes electrophysiological and structural changes that exacerbate or maintain AF [5]. This structural remodeling can contribute to both the development and maintenance of AF [6, 7]. Several factors associated with atrial structural remodeling have been identified [8], of which atrial fibrosis and myocyte hypertrophy are considered key [9].

Atrial fibrosis is a hallmark of arrhythmogenic structural remodeling [10, 11]. Cardiac fibrosis is defined as a detrimental process causing imbalanced extracellular matrix deposition and heart degradation. Cardiac fibrosis causes excessive fibroblast proliferation and accumulation of extracellular matrix proteins in the cardiac interstitial space [12]. Expansion of the extracellular matrix between cardiomyocytes may cause conduction delays and create alternate conduction pathways. These changes also result in ectopic foci and anisotropic conduction, creating nonuniform wave fronts that facilitate abnormal reentrant arrhythmias [13]. Unfortunately, the precise mechanisms of atrial remodeling in AF remain unclear.

Atrial structural remodeling consists primarily of collagens I and III, and the process of fibrosis is regulated by a cascade of fibro-proliferative signals, including TGF-β1 and angiotensin II [2, 14]. Calcineurin is a Ca^2+^-calmodulin-activated serine/threonine phosphatase that is ubiquitously expressed and plays an important role in transducing Ca^2+^-dependent signals. Calcineurin is a heterodimer comprising a calmodulin binding catalytic subunit A and a Ca^2+^ binding regulatory subunit B. Calcineurin is required for activation of transcription and induction of cell hypertrophy in a number of cell types including cardiac myocytes [15, 16]. Activation of calcineurin was reported to be increased in atrial tissues of patients with AF [17]. Calcineurin dephosphorylates NF-AT3 and NF-AT4, which induces their translocation from the cytosol to the nucleus where they activate the transcription of their target genes [17–19]. Transgenic mice that constitutively express calcineurin in cardiomyocytes can develop cardiac hypertrophy and a concomitant accumulation of collagen deposits surrounding the degenerating cardiomyocytes [19–21]. Calcineurin inhibitors such as cyclosporin A and FK506 can inhibit TGF-β1 expression to block increased extracellular matrix protein accumulation [22]. Furthermore, a constitutively active NF-AT 3 and NF-AT4 expressed in the hearts of transgenic mice produced cardiac wall fibrosis and cardiac myocyte enlargement, demonstrating the importance of a calcium-calcineurin-NF-AT3 pathway in cardiac fibrosis and hypertrophy [19].

The transcription factors NF-AT3 and NF-AT4 are the downstream effectors of calcineurin, and play an important role in the calcineurin-dependent pathway during cardiac hypertrophy [19, 23]. Recent studies also directly and indirectly implicate the calcineurin-dependent pathway in the development of cardiac fibrosis [19, 20, 24, 25]. In addition, the N-terminal propeptides of collagen types I or III (PINP and PIIINP, respectively) and the C-terminal propeptides (PICP) in the blood have been reported to be useful markers of collagen type I or III synthesis [26].

In the present study, we examined the correlation between nuclear NF-AT3 and NF-AT4 expression and distribution with collagens I and III expression levels in diseased atrial tissues of patients with AF. We also examined the correlations between serum levels of TGF-β1, PINP, PIIINP, and PICP with NF-AT3 and NF-AT4 expression in the nucleus and with collagen I and III expression in atrial tissues. In patients with AF, we found that the expression levels of nuclear NF-AT3 and NF-AT4 correlated with those of collagens I and III in the atria and with PICP and TGF-β1 in blood.

## Methods

### Patients

We recruited 90 VHD patients, comprising pathological changes in the mitral or aortic valves, or both, admitted to the First Affiliated Hospital of Nanjing Medical University for valve replacement surgery from January 2012 to January 2013. The patients were divided into three groups: sinus rhythm (SR; *n = 30*), persistent AF (PeAF; AF lasting >6 month, *n* = 30), and paroxysmal AF (PaAF; recurrent AF that terminated spontaneously in <7 days*, n* = 30). The control group (*n* = 10) comprised patients with congenital heart disease and SR who underwent heart surgery. We excluded four categories of patients from this study: (i) patients with renal dysfunction (serum creatinine >136 μmol/L) or Type II diabetes, (ii) patients whose coronary angiography and echocardiographic evaluation indicated coronary artery bypass grafting or associated procedures, (iii) patients >70 years, or those with a history of some diseases (e.g., hyperthyroidism) that influence AF risk, and (iv) patients with fibrosis disease that could affect serum fibrosis biomarkers. Preoperative medications, except warfarin and angiotensin-converting enzyme inhibitors, were continued until the morning of the surgery. Prior to surgery, an investigator assessed the preoperative clinical characteristics of the patients. Before discharge, another investigator recorded detailed operative data. The Ethics Committee of Nanjing Medical University approved the study protocol, and all patients provided written consent prior to enrollment. The investigation adhered to the principles outlined in the Declaration of Helsinki.

### Human cardiac tissue collection and storage

The same cardiac anesthesiologist, perfusionist, and surgical team performed all surgeries. All patients underwent cardiopulmonary bypass with moderate hypothermia (33–34°C). Antegrade crystalloid cardioplegia was used to arrest the heart, and local hypothermia was maintained with ice slush. A cardioplegic solution was readministered every 20–30 min. Approximately 250 mg of right atrial appendage (RAA) tissue was collected from the cannulation site, and approximately 250 mg of left atrial appendage (LAA) tissue was collected in the PeAF and PaAF group before initiating extracorporeal circulation. In our department, LAA ligation and resection is a routine surgical maneuver in rheumatic valvular disease patients with AF. To minimize damage, we only collected LAA samples from the AF group; this surgical maneuver was not necessary in the SR group. The sample site was similar because of similar surgical maneuvers. A 50 mg portion of RAA and LAA tissue was fixed in 4% paraformaldehyde for histology and immunohistochemistry. The remaining tissue was snap-frozen in liquid nitrogen for other analyses.

### Reverse transcription-polymerase chain reaction (RT-PCR)

Total RNA was isolated from the atrial tissue samples and treated with RNase-free water according to the TRIzol® (Invitrogen, Carlsbad, CA, USA) method. Single-stranded cDNA was synthesized from the total RNA as follows. In brief, 2 μg RNA was preincubated with 1.5 μL oligo (dT)_18_ primer (10 μmol/L; Genscript Technology Co., Nanjing, China), and diethylpyrocarbonate (DEPC)-treated water (0.1% DEPC; Keygen, China) in a total volume of 10 μL. This solution was incubated at 70°C for 10 min and then rapidly chilled on ice. The reaction was initiated by incubation at 42°C for 1 h in a Multigene™ Gradient TC9600-G-230 V thermal cycler (Labnet International Inc., Edison, NJ, USA) and was deactivated at 70°C for 15 min, followed by immersion in ice. The resultant cDNA was used as a template for subsequent PCR. Thirty cycles of PCR amplification were performed, with initial incubation at 94°C for 5 min and final extension at 72°C for 5 min. Each cycle comprised denaturation at 94°C for 30 s, annealing at 55°C for 30 s, and extension at 72°C for 30 s. The collagen I, collagen III, NF-AT3, and NF-AT4 genes were amplified using the following specific primers: *collagenI* (sense: 5′- TTCCTGCGCCTGATGTCC -3′, antisense: 5′- GGTTCAGTTTGGGTTGCTTGT -3′); *collagenIII* (sense: 5′- TCAACACCGATGAGATTATGAC -3′, antisense: 5′- CAAAGGATTGGCACTTATGC -3′); *NF-AT3* (sense: 5′- GGGACAACAGAACCAGAGTAAC -3′, antisense: 5′- AAACAGAATAGTCCACCTTGAGA -3′); *NF-AT4* (sense: 5′- TTGGAACACCAGCCATCAGG -3′, antisense: 5′- GCTGCTCCTGTTCTTTTGCC -3′). The quantities of cDNA that produced an equal amount of GAPDH PCR product were used for PCR using the primers for *collagenI, collagenIII*, *NF-AT3*, and *NF-AT4*. PCR product levels were semiquantitatively determined using a digital camera and an image analysis system (Gel Doc™ XR; Bio-Rad, Hercules, CA, USA), followed by normalization against GAPDH expression.

### Western blotting

In preparation of whole tissue extracts, atrial tissue samples were homogenized on ice in RIPA lysis buffer (Thermo Fisher Scientific Inc, Rockford, IL USA). Lysates were incubated on ice for 10 min at 4°C and subsequently centrifuged at 9300 × *g* for 10 min. Supernatants were saved and stored at -70°C. Nuclear extracts were prepared as follows [17]. Approximately 250 mg of atrial tissue was washed with cold PBS, homogenized, and resuspended in 1 ml of hypotonic buffer. Homogenates were incubated for 10 min on ice and centrifuged (10 min, 800 × *g* at 4°C). Pellets were resuspended in 0.15 ml of hypertonic buffer and incubated on ice for 20 min. Samples were centrifuged (10 min, 13 000 × *g* at 4°C), and supernatants (nuclear protein extract) were stored in aliquots at -80°C. Protein concentrations were determined using the Lowry method, and absorbance was measured spectrophotometrically (UV 2540; Shimadzu, Kyoto, Japan). Denatured samples were subjected to western blotting as follows. Samples containing 25 μg of protein were electrophoretically separated on a 10% SDS-polyacrylamide gel for 1.5 h at 120 V, and the proteins were transferred to nitrocellulose membranes (Pall Corporation, Ann Arbor, MI, USA). After blocking in 5% fat-free milk, the membranes were incubated overnight at 4°C with primary antibodies (dilution) against collagen I (1:200, Biosynthesis Biotechnology Company, Inc., Beijing, China), collagen III (1:200, Bioss), NF-AT3 [1:1000; Cell Signaling Technology Inc., USA), and NF-AT4 (1:1000; Cell Signaling Technology). Anti-GAPDH (1:1000; Cell Signaling Technology Inc.) and anti-lamin B (1:1000; Cell Signaling Technology Inc.) polyclonal antibodies were used as controls to normalize the data. The membranes were then incubated for 2 h at 37°C with secondary antibodies (goat anti-rabbit IgG diluted in PBS containing 5% fat-free milk and 0.1% Tween-20). The stained membranes were visualized using enhanced chemiluminescence with the ECL Plus reagent (GE Healthcare, Chalfont St. Giles, Buckinghamshire, UK). Western blotting was repeated at least thrice per sample with similar results.

### Blood sampling and enzyme-linked-immunosorbent serologic assay (ELISA)

Venous blood samples were obtained in EDTA from every patient before surgery. Serum was separated by centrifugation (1800 × g, 5 min, room temperature), and stored at -80°C until analysis. Serum PIIINP, PINP, TGF-β1, and PICP levels were determined by sensitive ELISA kits (Senxiong Biotechnology Industry Inc., Shanghai, China), according to the manufacturer’s instructions. Assays were performed in duplicate in a single run and normalized to a standard curve.

### Histology and immunohistochemistry

RAA and LAA samples were fixed with 4% paraformaldehyde in phosphate-buffered saline (pH 7.4) for 24 h. After alcohol dehydration, the tissues were embedded in paraffin and sectioned. The 2-μm-thick serial sections were then stained with Van Gieson’s solution for microscopic examination. For NF-AT3 and NF-AT4 (Biosis) detection, immunoreactivity was performed on 4-μm-thick sections of the paraffin-embedded tissues. Brown staining in the cells or cell membranes was considered positive. Hypertrophic heart ventricle tissues were selected as a positive control, and negative controls were sections incubated with antibodies pre-absorbed with the NF-AT3 and NF-AT4 peptide (Abcam). The entire sections were scanned at low magnification (×100) initially to select regions, and then high magnification (×400) was used for focused investigation.

### Statistical analyses

Values are expressed as the mean ± standard deviation. Differences among three or more groups were analyzed using the Kruskal–Wallis test. Differences between any two groups were analyzed using the Mann–Whitney *U* test. Chi-square and Fisher’s exact tests were used to determine the differences between the groups. Univariate regression tests were used to assess the associations among the expression of NF-AT3 and NF-AT4 in the nucleus, collagens I and III in atrial tissue, and PINP, PIIINP, PICP, and TGF-β1 in the blood. Differences yielding p <0.05 were considered significant. Data were analyzed using GraphPad Prism version 5.01 and STATA version 10.0.530.0.

## Results

### Clinical characteristics and hemodynamic data

Preoperative hemodynamic and echocardiographic data are shown in Table 1. Statistical analysis showed that left atrial diameter, measured by echocardiography, was significantly larger in the PeAF group than in the SR group. Furthermore, the left atrial diameter was significantly larger than the right atrial diameter in all groups, except for the control group. Right atrial diameters, left ventricular end systolic dimensions (LVDs), and left ventricular end diastolic dimensions were not significantly different among the three VHD groups. The constituent ratios of patients with different heart functions were not significantly different among the three VHD groups.

**Table 1:** **Analysis of clinical data**

	SR + CHD	SR + VHD	PaAF + VHD	PeAF + VHD
Patient number	10	30	30	30
Sex, M/F (n)	4/6	16/14	13/17	12/18
Age (years)	16.20 ± 3.31	54.75 ± 3.68	55.61 ± 6.83	53.58 ± 4.63
Preoperative data				
Heart rate (beats/min)	102.4 ± 4.51	79 ± 6.00	81 ± 7.00	83 ± 9.20
NYHA class I/II/III/IV	2/7/1/0	0/12/10/8	0/7/13/10	0/5/12/13
Echocardiographic parameters				
LVDd	34 ± 2.30	53.55 ± 8.70	55.66 ± 14.13	54.97 ± 12.36
LVDs	24 ± 3.11	36.38 ± 1.26	39 ± 10.22	34.13 ± 11.03
EF (%)	64 ± 4.12	55.01 ± 4.80	53.08 ± 5.32	52.21 ± 5.21
LAD (mm)	23 ± 6.73	47.25 ± 5.19^c^	55.43 ± 6.80^a,c^	59.23 ± 5.92^b,c^
RAD (mm)	27 ± 8.71	38.40 ± 5.20	38.10 ± 4.64	38.20 ± 5.00
Preoperative length of stay	10.00 ± 9.36	15.00 ± 1.80	16.00 ± 11.53	16.00 ± 13.40
Valve disease				
Pure MVD	0	12	15	12
Mitral stenosis		4	5	6
Mitral regurgitation		5	6	2
Mitral stenoregurgitation		3	4	4
Pure AVD	0	10	6	5
Aortic valve stenosis		6	3	1
Aortic valve regurgitation		4	3	4
DVD	0	8	9	13
Preoperative drug				
Digitalis	0	18	28	30
Diuretic	4	22	24	28
Nitrate drug	0	26	23	27
Calcium channel blocker	0	10	12	11
β-blocker	3	10	22	30
ACEI	0	19	21	18
Operative data				
Surgical procedure				
MVR/AVR/DVR	0/0/0	12/10/8	15/6/9	12/5/13
CPB duration	78 ± 11.44	130 ± 12.42	141 ± 23.44	145 ± 13.23
Aortic clamp time	45.32 ± 33.32	79 ± 34.43	82 ± 35.26	90 ± 42.13

Data are presented as n or mean ± standard deviation.

AVD, aortic valve disease; CHD, Congenital heart disease; DVD, Double valvular disease (mitral and aortic valves); MVD, Mitral valvular disease; PaAF, Paroxysmal atrial fibrillation; PeAF, Persistent atrial fibrillation; SR, Sinus rhythm; VHD, Valvular heart disease.

^a^PaAF + VHD vs. SR + VHD; P < 0.01. ^b^PeAF + VHD vs. SR + VHD; P < 0.01. ^c^LAD vs. RAD; P < 0.01.

### Serum PINP, PIIINP, PICP, and TGF-β1 levels were increased in the AF groups

The levels of PICP and TGF-β1 were significantly increased in the PeAF group compared with the PaAF and SR groups. However, there were no differences in PIIINP levels between the PeAF and PaAF groups, while PIIINP levels in the PeAF group were only significantly higher than those in the SR group. Levels of PINP in the PeAF group were significantly greater than those in the control group, while there were no differences in PINP levels between the PeAF and the other groups (Table 2).

**Table 2:** **PIIINP, PINP, PICP, and TGF-β1 blood levels**

	PeAF + VHD (n = 30)	PaAF + VHD (n = 30)	SR + VHD (n = 30)	SR + CHD (n = 10)
PIIINP (ng/ml)	88.03 ± 46.08^b,c^	72.96 ± 43.30^d^	52.50 ± 29.32^e^	14.09 ± 2.732
PINP (ng/ml)	45.41 ± 42.17^c^	34.82 ± 28.60	18.95 ± 17.34	6.940 ± 3.007
TGF-β1 (pg/ml)	49.01 ± 28.67^a,b,c^	27.96 ± 13.16^d^	24.09 ± 14.10	11.43 ± 2.695
PICP (ng/ml)	43.42 ± 3.699^a,b,c^	36.06 ± 7.710^d^	29.75 ± 9.241^e^	12.52 ± 3.033

Data are presented as n or mean ± standard deviation.

CHD, Congenital heart disease; PaAF, Paroxysmal atrial fibrillation; PeAF, Persistent atrial fibrillation; PICP, carboxyl-terminal peptide from pro-collagen I; PINP, N-terminal type I procollagen propeptides; PIIINP, N-terminal type III procollagen propeptides; SR, Sinus rhythm; VHD, Valvular heart disease.

^a^PeAF + VHD vs. PaAF + VHD; P < 0.05. ^b^PeAF + VHD vs. SR + VHD; P < 0.05. ^c^PeAF + VHD vs. SR + CHD; P < 0.05. ^d^PaAF + VHD vs. SR + CHD; P < 0.05. ^e^SR + VHD vs. SR + CHD; P < 0.05.

### Expression of collagen I, collagen III, NF-AT3, and NF-AT4 mRNA and protein were increased in the AF groups

**Figure 1:**
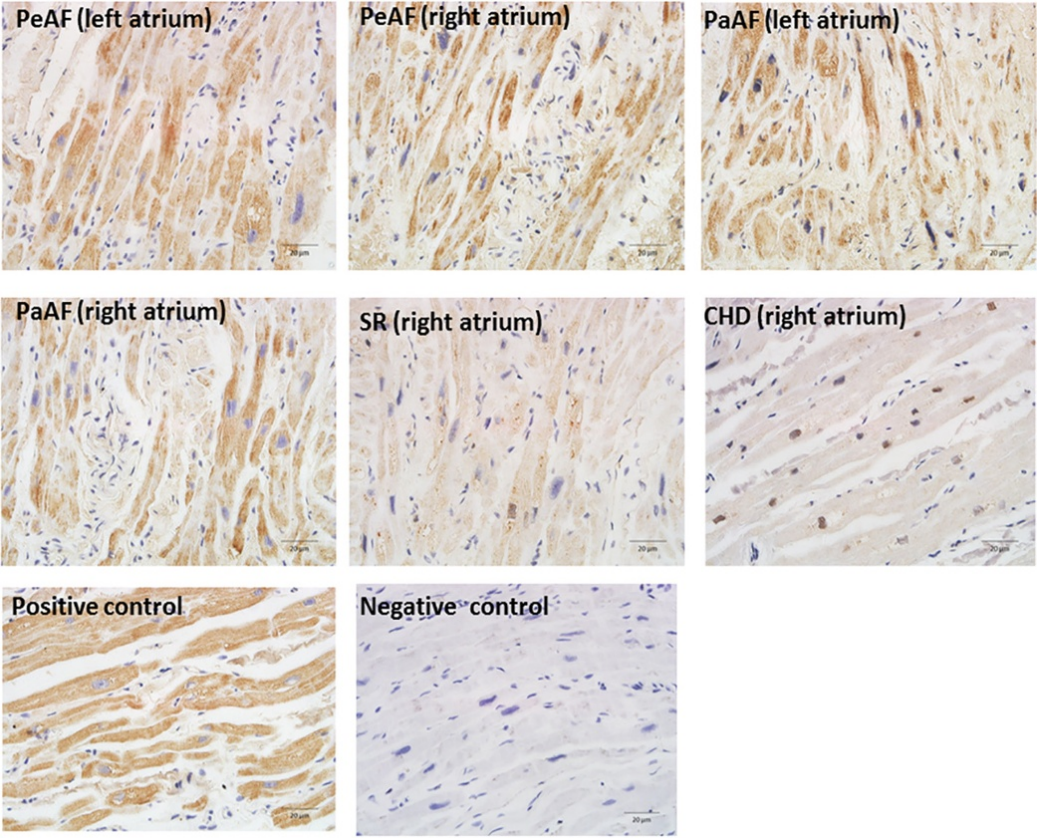
**Immunohistochemistry for NF-AT3 (stained brown) in sections obtained from the CHD (right atrium), SR (right atrium), PaAF (right atrium), PaAF (left atrium), PeAF (right atrium), and PeAF (left atrium) groups.** Nuclei are in blue. Elevated levels of NF-AT3 in the right and left atrial tissues were identified in the PeAF groups compared with the SR and CHD groups. Hypertrophic heart ventricle tissues were selected as a positive control, and negative controls were sections incubated with antibodies pre-absorbed with the NF-AT3 and NF-AT4 peptide. Magnification (×400). CHD, Congenital heart disease; SR, Sinus rhythm; PaAF, Paroxysmal atrial fibrillation; PeAF, Persistent atrial fibrillation; NF-AT3, Nuclear factor of activated T cells 3; NF-AT4, Nuclear factor of activated T cells 4.

**Figure 2:**
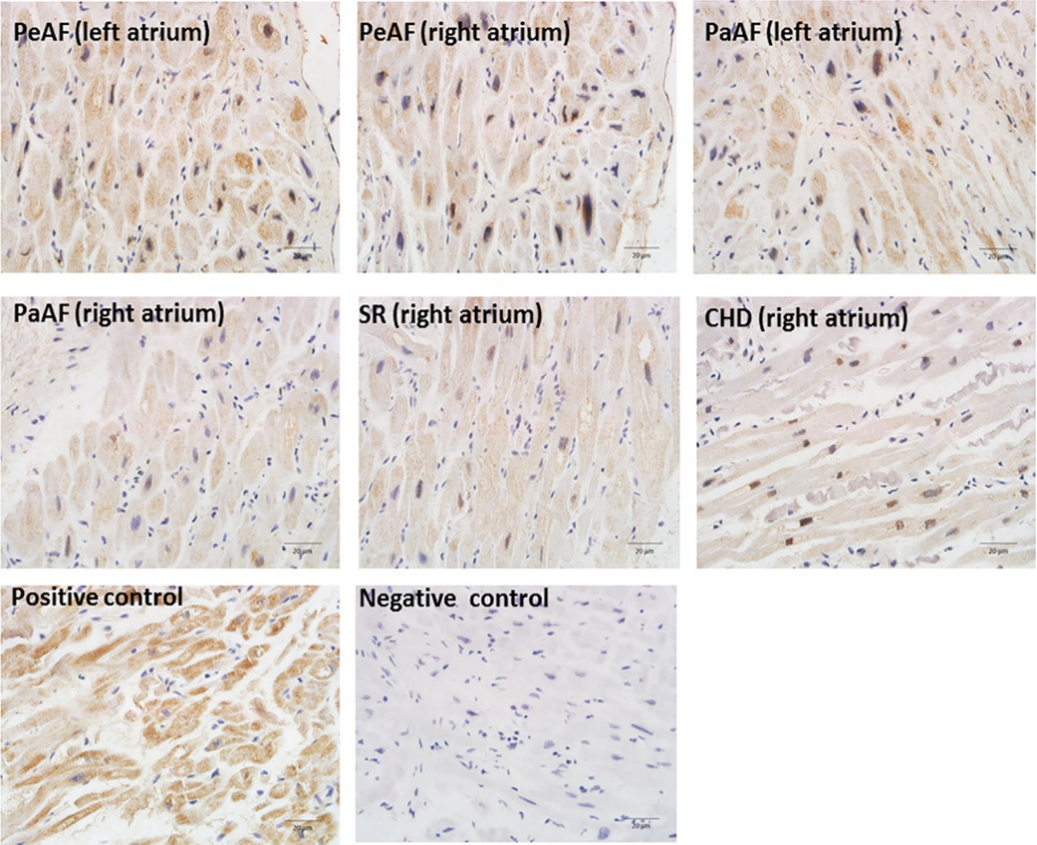
**Immunohistochemistry for NF-AT4 (stained brown) in sections obtained from the CHD (right atrium), SR (right atrium), PaAF (right atrium), PaAF (left atrium), PeAF (right atrium), and PeAF (left atrium) groups.** Nuclei are in blue. Elevated levels of NF-AT3 in the right and left atrial tissues were identified in the PeAF groups compared with the SR and CHD groups. Hypertrophic heart ventricle tissues were selected as a positive control, and negative controls were sections incubated with antibodies pre-absorbed with the NF-AT3 and NF-AT4 peptide. Magnification (×400). CHD, Congenital heart disease; NF-AT3, Nuclear factor of activated T cells 3; NF-AT4, Nuclear factor of activated T cells 4; PaAF, Paroxysmal atrial fibrillation; PeAF, Persistent atrial fibrillation; SR, Sinus rhythm.

**Figure 3:**
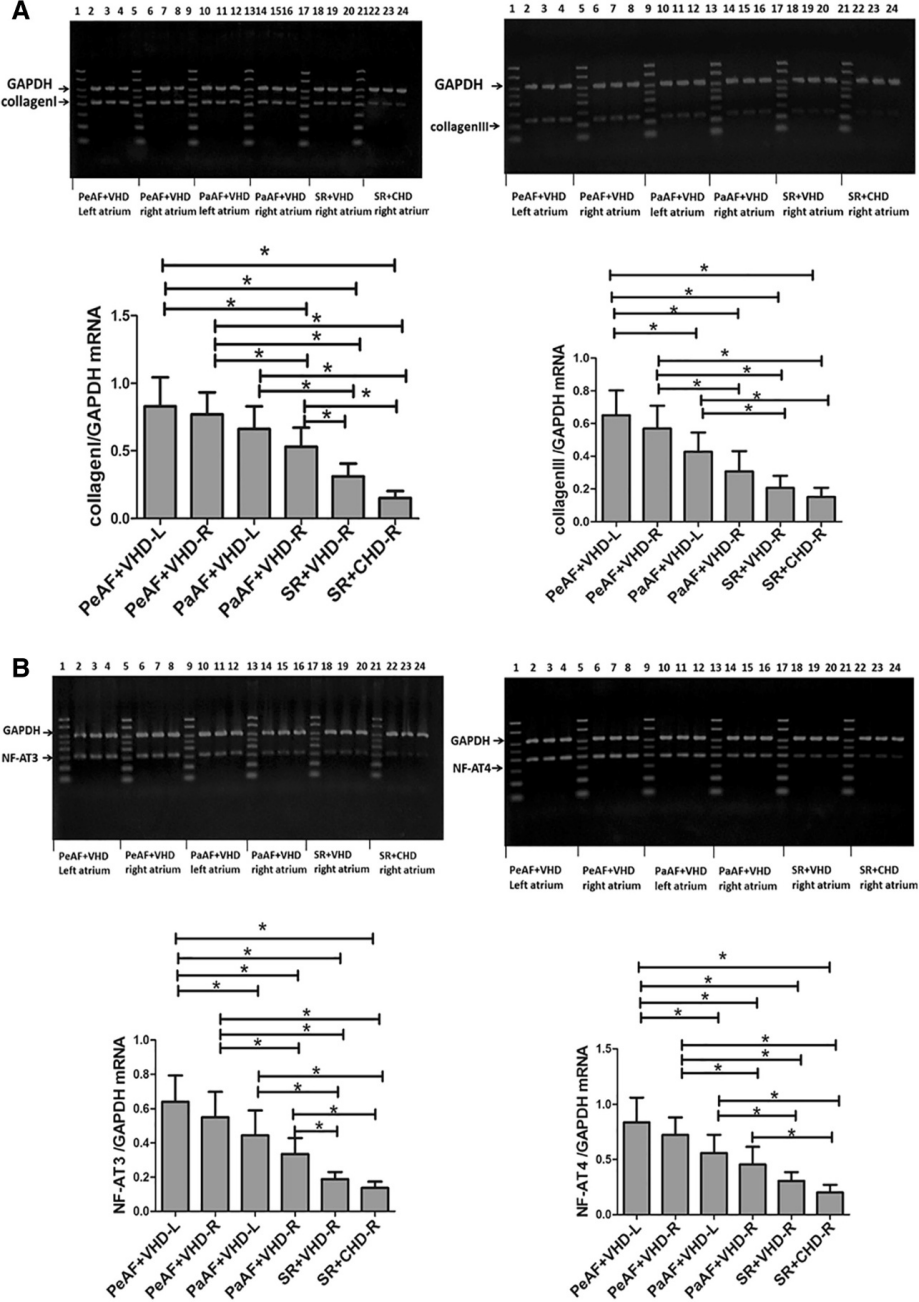
**Collagen I,collagen III,NF-AT3 and NF-AT4 mRNA expression in the different groups** **(A)** **Collagen I and collagen III mRNA expression in the different groups.** *P < 0.05. **(B)** NF-AT3 and NF-AT4 mRNA expression in the different groups. *P < 0.05. GAPDH indicates a representative blot. NF-AT3, Nuclear factor of activated T cells 3; NF-AT4, Nuclear factor of activated T cells 4.

**Figure 4:**
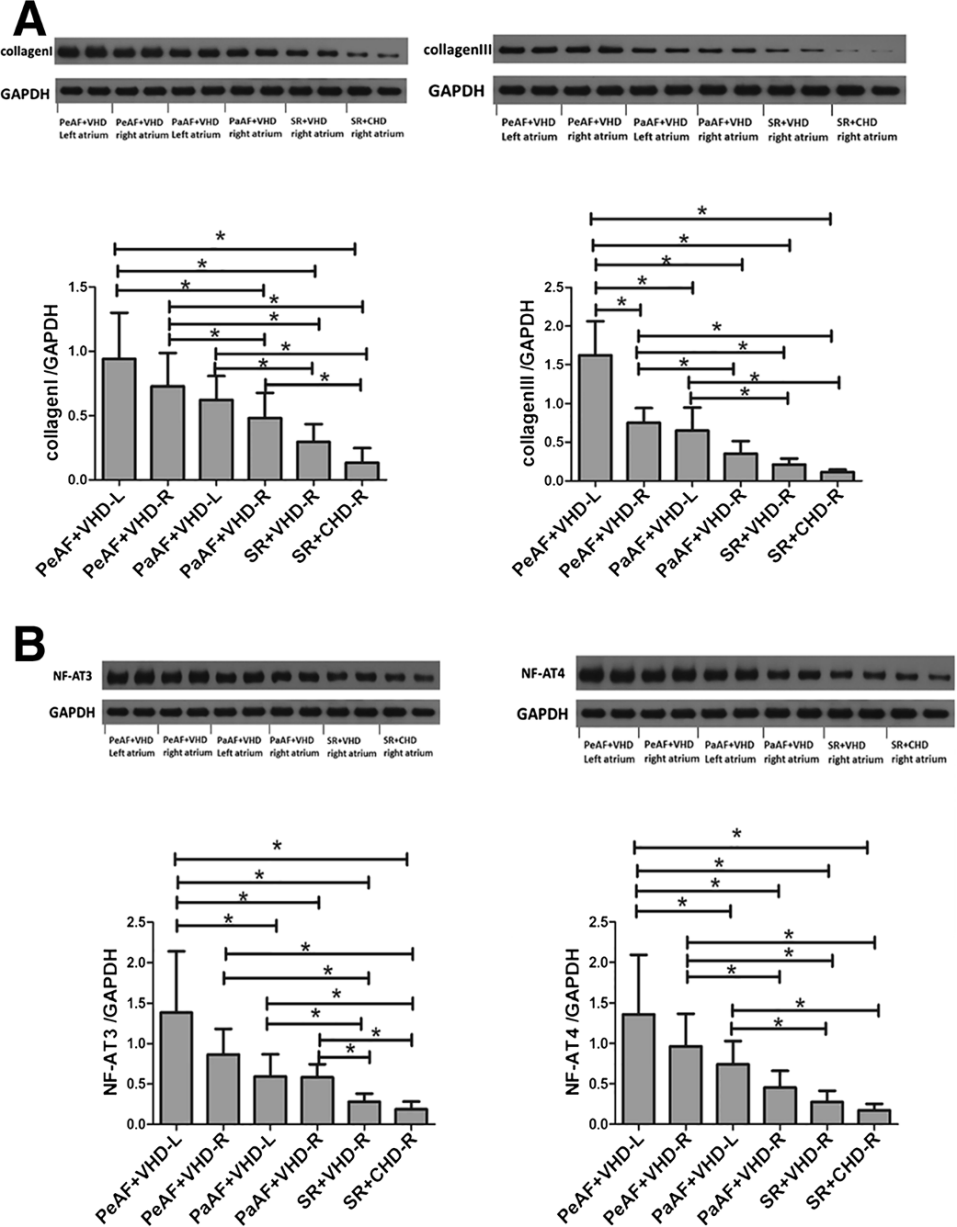
**Collagen I,collagen III,NF-AT3 and NF-AT4 protein expression in the different groups** **(A)** **Collagen I and collagen III protein expression in the different groups.** P < 0.05. **(B)** NF-AT3 and NF-AT4 protein expression in the different groups. *P < 0.05. GAPDH indicates a representative blot. NF-AT3, Nuclear factor of activated T cells 3; NF-AT4, Nuclear factor of activated T cells 4.

**Figure 5:**
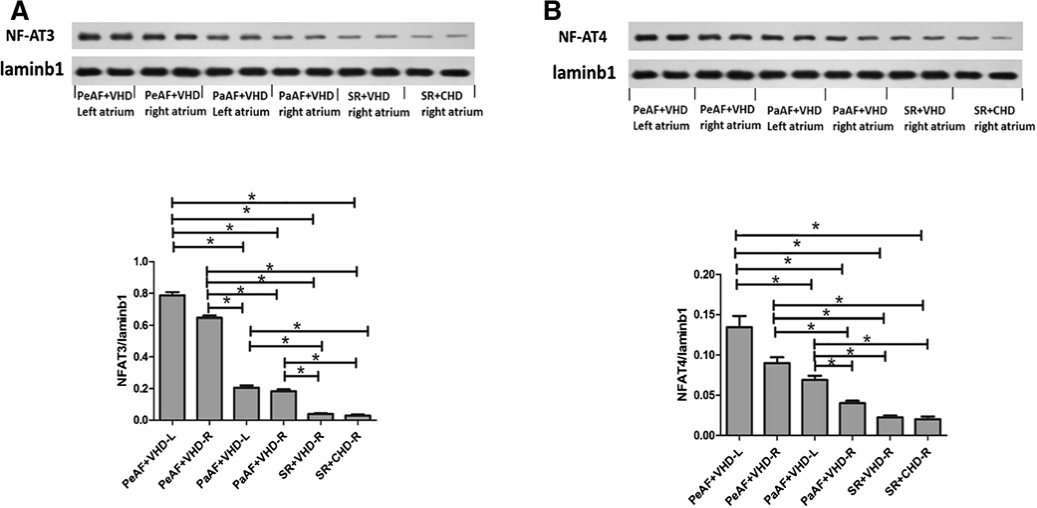
**Nuclear NF-AT3 and Nuclear NF-AT4 protein expression in the different groups** **(A)** **Nuclear NF-AT3 protein expression in the different groups.** *P < 0.05. **(B)** Nuclear NF-AT4 protein expression in the different groups. *P < 0.05. Laminb1 indicates a representative blot. NF-AT3, Nuclear factor of activated T cells 3; NF-AT4, Nuclear factor of activated T cells 4.

Immunohistochemistry showed that total NF-AT3 (Figure 1) and NF-AT4 (Figure 2) expression were upregulated in the AF groups compared with the SR and control groups. We also demonstrated that collagen I, collagen III, total NF-AT3, and NF-AT4 expression were upregulated in the AF groups using RT-PCR and western blotting (Figure 3A,B; Figure 4A,B). We also demonstrated that nuclear NF-AT3 and NFAT4 expression was upregulated in the AF group compared with the SR group (Figure 5A,B).

### Analysis of the expression of NF-AT3 and NF-AT4 and collagens I and III in the right and left atria of patients with AF

In the left atrium of patients with AF, collagen I mRNA expression was not correlated with NF-AT3 and NF-AT4 mRNA expression. Collagen I protein expression was positively correlated with nuclear NF-AT3 expression (p <0.01), but not with nuclear NF-AT4 expression. Levels of collagen III mRNA expression were positively correlated with NF-AT4 mRNA expression (p < 0.01), but not with NF-AT3 expression. Collagen III protein expression was positively correlated with nuclear NF-AT3 (p <0.01) and nuclear NF-AT4 expression (p <0.01).

In the right atrium of patients with AF, collagen I mRNA expression was correlated with NF-AT3 (p <0.01) and NF-AT4 mRNA expression (p <0.01). Collagen I expression was correlated with nuclear NF-AT3 expression (p <0.01), but not with nuclear NF-AT4 expression. Collagen III mRNA expression was correlated with NF-AT3 (p <0.01) and NF-AT4 mRNA expression (p <0.01). Collagen III expression was correlated with nuclear NF-AT3 expression (p <0.01) and nuclear NF-AT4 (p <0.01) (Table 3).

**Table 3:** **Correlation between expression of collagens and nuclear NF-AT3 and NF-AT4 in the left and right atria of patients with atrial fibrillation mRNA**

		NF-AT3	NF-AT4
Collagen I	Left atrium	NF-AT3 mRNA	NF-AT4 mRNA
mRNA		Not significant	Not significant
	Right atrium	NF-AT3 mRNA	NF-AT4 mRNA
		r = 0.351, P < 0.01	r = 0.412, P < 0.01
Collagen III	Left atrium	NF-AT3 mRNA	NF-AT4 mRNA
mRNA		Not significant	r = 0.354, P < 0.01
	Right atrium	NF-AT3 mRNA	NF-AT4 mRNA
		r = 0.526, P < 0.01	r = 0.420, P < 0.01
		**NF-AT3**	**NF-AT4**
Collagen I	Left atrium	NF-AT3 protein	NF-AT4 protein
protein		r = 0.447, P < 0.01	No significant
	Right atrium	NF-AT3 protein	NF-AT4 protein
		r = 0.469, P < 0.01	Not significant
Collagen III	Left atrium	NF-AT3 protein	NF-AT4 protein
protein		r = 0.774, P < 0.01	r = 0.421, P < 0.01
	Right atrium	NF-AT3 protein	NF-AT4 protein
		r = 0.740, P < 0.01	r = 0.404, P < 0.01

NF-AT3, Nuclear factor of activated T cells 3; NF-AT4, Nuclear factor of activated T cells 4.

### Correlation between collagen I and collagen III levels in the right and left atria with PIIINP, PINP, TGF-β1, and PICP levels in the blood of patients with AF

In the left atrium of patients with AF, collagen I levels were correlated with PICP (p = 0.001) and PINP levels (p = 0.003), but not with TGF**-**β1 levels. The level of collagen III was correlated with PIIINP (p <0.001) and TGF**-**β1 levels (p = 0.0042). Collagen I levels were correlated with PICP (p = 0.019) and TGF**-**β1 levels (p <0.05), while there was no correlation of collagen III levels with PIIINP and TGF**-**β1 levels in the right atrium of patients with AF (Table 4).

**Table 4:** **Correlation between serum fibrosis biomarkers and collagen levels in the right and left atria of patients with atrial fibrillation**

		Fibrosis biomarker	
Collagen I	Left atrium	PICP	PINP
		r = 0.386, P = 0.001	r = 0.423, P = 0.003
	Right atrium	PICP	TGF-β1
		r = 0.336, P = 0.019	r = 0.258, P < 0.05
Collagen III	Left atrium	TGF-β1	PIIINP
		r = 0.469, P < 0.001	r = 0.291, P = 0.042
	Right atrium	Not significant	Not significant

PICP, carboxyl-terminal peptide from pro-collagen I; PINP, N-terminal type I procollagen propeptides; PIIINP, N-terminal type III procollagen propeptides.

### Correlation between nuclear NF-AT 3 and NF-AT 4 levels in the right and left atria and PIIINP, PINP, TGF-β1, and PICP levels in the blood of patients with AF

In the left atrium of patients with AF, nuclear NF-AT3 levels were correlated with PICP (p <0.01) and TGF**-**β1 levels (p <0.01), while nuclear NF-AT4 levels correlated with PICP (p <0.01) and TGF**-**β1 levels (p <0.05). In the right atrium of patients with AF, nuclear NF-AT3 levels were correlated with PICP (p <0.01) and TGF-β1 levels (p <0.01), while nuclear NF-AT4 levels correlated with PINP (p <0.01) and PICP levels (p <0.05 (Table 5).

**Table 5:** **Correlation between markers of serum fibrosis and levels of nuclear NF-AT3 and NF-AT4 in the right and left atria of patients with atrial fibrillation**

		Fibrosis biomarker	
Nuclear	Left atrium	PICP	TGF-β1
NF-AT3		r = 0.538, P < 0.01	r = 0.444, P < 0.01
	Right atrium	PICP	TGF-β1
		r = 0.538, P < 0.01	r = 0.387, P < 0.01
Nuclear	Left atrium	PICP	TGF-β1
NF-AT4		r = 0.282, P < 0.01	r = 0.324, P < 0.05
	Right atrium	PICP	PINP
		r = 0.264, P < 0.05	r = 0.316, P < 0.01

NF-AT3, Nuclear factor of activated T cells 3; NF-AT4, Nuclear factor of activated T cells 4; PICP, carboxyl-terminal peptide from pro-collagen I; PINP, N-terminal type I procollagen propeptides.

### Analysis of NF-AT3 and NF-AT4 protein expression in the right atria of patients with different valvular disease

In the right atrium, the total NF-AT3 expression in patients in the AF group was higher than that in patients in the SR group with mitral valve disease (MVD). NF-AT3 expression in patients in the PeAF group was higher than that in patients in the PaAF and SR groups with double-valve disease (DVD) (p <0.05). The total NF-AT3 expression in patients in the AF group was higher than that in those in the SR group with aortic valve disease (AVD) (p <0.05). NF-AT3 expression in patients with MVD and DVD was higher than those with AVD in the PeAF group, while NF-AT3 expression in patients with MVD was higher than in those with DVD or AVD in the PaAF group (p <0.05). NF-AT3 expression in patients with MVD was higher than those with DVD or AVD in the SR group (p <0.05). NF-AT4 expression in patients in the PeAF group was higher than those in the PaAF and SR groups with MVD, while NF-AT4 expression in the PeAF group was higher than those in the PaAF and SR groups with DVD (p <0.05). NF-AT4 expression in patients in the PeAF group was higher than those in the SR group with AVD (p <0.05). NF-AT4 expression in patients with MVD and DVD was higher than those with AVD in the PeAF group, while NF-AT4 expression in patients with MVD was higher than those with AVD in the PaAF group (p <0.05). NF-AT4 expression in patients with MVD and DVD was higher than those with AVD in the SR group (p <0.05; Table 6).

**Table 6:** **Protein expression of total NF-AT3 and NF-AT4 in right atria with different valvular disease in different group**

**NF-AT3**			
	**PeAF group (n = 30)**	**PaAF group (n = 30)**	**SR group (n = 30)**
MVD	0.990 ± 0.398^a,b,g^ (n = 12)	0.695 ± 0.147^i,j^ (n = 15)	0.313 ± 0.109^k,l^ (n = 12)
DVD	0.880 ± 0.183^c,d,h^ (n = 13)	0.516 ± 0.053 (n = 9)	0.331 ± 0.050 (n = 10)
AVD	0.522 ± 0.039^e,f^ (n = 5)	0.396 ± 0.048 (n = 6)	0.170 ± 0.071 (n = 8)
**NF-AT4**			
	**PeAF group (n = 30)**	**PaAF group (n = 30)**	**SR group (n = 30)**
MVD	1.109 ± 0.375^a,b,f^ (n = 12)	0.559 ± 0.222^h^ (n = 15)	0.332 ± 0.147^i^ (n = 12)
DVD	1.010 ± 0.379^c,d,g^ (n = 13)	0.415 ± 0.112 (n = 9)	0.331 ± 0.132^j^ (n = 10)
AVD	0.492 ± 0.109^e^ (n = 5)	0.244 ± 0.054 (n = 6)	0.158 ± 0.018 (n = 8)

Data are presented as n or mean ± standard deviation.

AVD, aortic valve disease; MVD, Mitral valvular disease; DVD, Double valvular disease (mitral and aortic valves); PaAF, Paroxysmal atrial fibrillation; PeAF, Persistent atrial fibrillation; SR, Sinus rhythm.

^a^MVD + PeAF group vs. MVD+ SR group; P < 0.05. ^b^MVD + PaAF group vs. MVD + SR group; P < 0.05. ^c^DVD + PeAF group vs. DVD + SR group; P < 0.05. ^d^DVD + PeAF group vs. DVD + PaAF group; P < 0.05. ^e^AVD + PeAF group vs. AVD + SR group; P < 0.05. ^f^AVD + PaAF group vs. AVD + SR group P < 0.05. ^g^MVD + PeAF group vs. AVD + PeAF group; P < 0.05. ^h^DVD + PeAF group vs. AVD + PeAF group; P < 0.05. ^i^MVD + PaAF group vs. AVD + PaAF group; P < 0.05. ^j^MVD + PaAF group vs. DVD + PaAF group P < 0.05. ^k^MVD + SR group vs. AVD + SR group; P < 0.05. ^l^DVD + SR group vs. AVD + SR group; P < 0.05. DVD: Double valve disease; MVD: Mitral valve disease; AVD: Aortic valve disease.

## Discussion

Atrial structural remodeling underlies AF development [8]. AF can cause structural remodeling that can exacerbate or maintain AF [5], while valvular disease also can induce atrial remodeling. However, the precise mechanisms of atrial remodeling in AF remain unclear. The development of sensitive serum biomarkers reflecting atrial remodeling may provide a simple method to determine the presence of atrial remodeling, allowing initiation of timely therapeutic interventions. In the present study, we determined that total NF-AT3 and NF-AT4 expression was increased in the PeAF group (particularly in the left atrium) and the AF group.

As dilated atria caused by valvular disease can affect atrial fibrosis and induce expression of related proteins, comparisons of left atria between the PeAF and SR groups may be confounded differences in left atrial sizes. By contrast, right atrial diameters were not significantly different between the three groups, and were thus considered suitable for studying the direct effects of AF on atrial remodeling. In the right atria, we found that NF-AT3 and NF-AT4 expression was increased in the PeAF group compared with that in the SR group with similar valvular disease. Moreover, NF-AT3 and NF-AT4 expression in patients with pure mitral valvular disease was increased compared with that in patients with pure aortic valvular disease in the PeAF, PaAF, and SR groups. These data suggest that expression of NF-AT3 and NF-AT4 are altered by atrial fibrillation and valvular disease (particularly MVD). Thus, atrial fibrillation may be an important factor affecting NF-AT3/4 expression and atrial remodeling.

We also found a significant relationship between the expression of collagen and nuclear NF-AT3 and NF-AT4 (Table 6). Collagens I and III are secreted as pro-collagen precursors containing amino- and carboxyl-terminal propeptides, which are released into the serum by proteases after collagen deposition [27]. The N-terminal propeptides of collagen I or III (PINP and PIIINP, respectively) and the C-terminal propeptides (PICP) are used as markers of collagen type I or III synthesis [26]. We demonstrated that serum PINP, PIIINP, and PICP were increased in the PeAF group. Final analyses showed that PINP (PIN, r = 0.423 > PICP, r = 0.386) and TGF-β1 (TGF-β1, r = 0.469 > PIIINP, r = 0.291) were sensitive fibrosis biomarkers for the left atria of AF patients, while PICP (PICP, r = 0.336 > TGF-β1, r = 0.258) was a sensitive fibrosis biomarker for the right atria. To establish the extent of right and left atrial fibrosis, PICP and TGF-β1 are likely the most sensitive fibrosis biomarkers. We also found that serum PICP and TGF-β1 levels were the optimal biomarkers for indicating the effect of nuclear NF-AT3 and NF-AT4 on atrial fibrosis in patients with AF. Nuclear NF-AT3 expression in the right and left atria correlated significantly with PICP and TGF-β1 levels in the blood of patients with AF. Nuclear NF-AT4 levels in left atria correlated significantly with PICP and TGF-β1 levels, while those in the right atria correlated significantly with PICP and PINP levels in the blood of patients with AF. Therefore, we conclude that PICP and TGF-β1 may be useful serum fibrosis biomarkers to estimate the extent of atrial remodeling due to atrial fibrillation or other risk factors.

## Conclusion

We demonstrate that NF-AT3 and NF-AT4 expression were increased in patients with atrial fibrillation. Furthermore, nuclear NF-AT3 and NF-AT4 expression were correlated significantly with levels of collagen I and III l in the atrium and with PICP and TGF-β1 levels in the blood. These data support the hypothesis that nuclear NF-AT3 and NF-AT4 participates in atrial structural remodeling, and that PICP and TGF-β1 levels may be sensitive serum biomarkers to estimate atrial structural remodeling. However, because there are many risk factors for AF, the mechanism of NF-AT3 and NF-AT4 expression in atrial structural remodeling requires further investigation.

## Abbreviations

AF: Atrial fibrillation
AVD: Aortic valve disease
CHD: Congenital heart disease
DVD: Double valvular disease (mitral and aortic valves)
MVD: Mitral valvular disease
NF-AT3: Nuclear factor of activated T cells 3
NF-AT4: Nuclear factor of activated T cells 4
PaAF: Paroxysmal atrial fibrillation
PeAF: Persistent atrial fibrillation
PICP: Carboxyl-terminal peptide from pro-collagen I
PIIINP: N-terminal type III procollagen propeptides
PINP: N-terminal type I procollagen propeptides
SR: Sinus rhythm
VHD: Valvular heart disease.
